# 

**Published:** 1889-04-06

**Authors:** 


SATURDAY, APRIL 6th, 1889.

**Examinations in Elementary Schools** - - - 1

**Turning over New Leaves.**—Guide to Bearer and Stretcher Company Drill - - - - - - 2

**The Doctor's Pulpit.**—Man and his Enemies - - 3

**Within the Wards.**—Vivisectors and their Work - - 4

**Opium and its Votaries.**—By Sir William Moore - 5

**The Student's Column.**—"A Text-book of Pathological Anatomy and Pathogenesis" - - - - - - - 6

**How to give Medicine to Children.**—By C. R. Illingworth, M.D. - - - - - - - - 7

**Notes and News** - - - - - - - - - 8

**Everybody's Page** - - - - - - - - 10

**Annotations.**—The Children of Humanity—Leisure and Temper—The Burden of the Books - - - - 11

**A Street Ambulance Service for London** - - - 12

**"Her Heart's Mistake."**—By E. D. Cumming, Author of "An Ordeal by Silence" - - - - - - 13

**Abstainers and Life Insurance** - - - - - 15

**Scraps and Gleanings** - - - - - - - 16

**Amusements and Relaxation** - - - - - 16

**EXTRA SUPPLEMENT—THE NURSING MIRROR.**

**En Passant.**—An Entertainment for Nurses—Advertisements—Edinburgh School of Medicine—A Brave Nurse—Lady Nurses—Institution News—The Teaching of Nurses i
**The Nursing and Management of the Insane.**—By T. Duncan Greenlees, M.B. - - - - - - ii
**Notes from Australia** - - - - - - - - ii
**Nurses in the Soudan.**—The Start from England - - iii
**Everybody's Opinion.**—Combination of Private Nurses—Wanted: Suggestions for a Peaceful Night—Private Nurses—The Food Question - - - - - - - iv
**Appointments—Vacancies—Notes and Queries** - iv
**Presentation—The Nurse's Case Book** - - - iv

